# Fertility outcomes after uterine artery embolization for symptomatic leiomyomas

**DOI:** 10.1186/s42155-025-00604-4

**Published:** 2025-10-16

**Authors:** Coralie Fabre, Tom Boeken, Vanille Simon, Carole Dean, Marc Sapoval, Olivier Pellerin, Anne-Sophie Bats, Henri Azaïs, Meriem Koual

**Affiliations:** Hôpital Europeen Georges-Pompidou: Hopital Europeen Georges Pompidou, Paris, France

**Keywords:** Leiomyoma, Uterine fibroids, Uterine Artery Embolization, Fertility, Pregnancy

## Abstract

**Background:**

Uterine artery embolization (UAE) is a recognized treatment for symptomatic fibroids, but its impact on fertility remains controversial. This study aimed to assess live birth rates, pregnancy outcomes, and obstetric complications in patients attempting pregnancy after UAE.

**Materials and methods:**

We conducted a retrospective monocentric study including women aged 18–45 years treated by UAE for symptomatic fibroids between June 2007 and March 2021. Patients who attempted pregnancy post-procedure were identified and analyzed. The primary outcome was the live birth rate; secondary outcomes included pregnancy rate and obstetric complications. Statistical analyses were performed according to the STROBE guidelines.

**Results:**

Among 210 included patients, 46 attempted pregnancy (22%). The mean age of this fertility population was 40 years. Thirteen women (28%) began at least one pregnancy after UAE, resulting in 12 births overall in 9 mothers. All live births were delivered via cesarean section, and no fatal obstetric complications occurred in this cohort. The miscarriage rate (23%) and other obstetric outcomes were consistent with general population trends for similar age groups. UAE demonstrated high symptom resolution, with 70% of patients requiring no further interventions for fibroids.

**Conclusion:**

Fertility may be preserved in a subset of women with complex surgical histories or high-risk surgical profiles undergoing UAE for symptomatic fibroids.

**Trial registration:**

NCT05271981

## Introduction

Uterine leiomyomas, also known as fibroids, are the most common benign tumors in women of childbearing age, with a prevalence increasing with age until menopause, potentially reaching 70% [1]. Although often asymptomatic, they can cause heavy menstrual bleeding, pelvic pain, dyspareunia, and infertility, significantly impacting quality of life [2]. Leiomyomas are associated with reduced fertility, with studies indicating a 10–30% lower pregnancy rate in affected women compared to age-matched controls, particularly due to distorted uterine anatomy and impaired endometrial receptivity [3]. Infertility risks escalate with advancing age, as women over 35 face a natural decline in ovarian reserve and a higher prevalence of leiomyomas, compounding conception challenges [4].

Several fertility-preserving options are available for symptomatic women, including medical treatments, selective myomectomy, high-intensity focused ultrasound (HIFU), and uterine artery embolization (UAE). Uterine artery embolization, developed in the 1990s and refined in the 2000s through animal and clinical studies, has been a key treatment for symptomatic women for over three decades, achieving symptomatic relief rates exceeding 85% with low complication rates, short hospitalization times, and quick recovery, as supported by numerous randomized trials [5–10].

For patients with large polymyomatous uteri and numerous leiomyomas, surgery is challenging and carries a high risk of complications, making UAE a valuable less invasive alternative. However, past studies were cautious about UAE for women desiring future pregnancies due to concerns about fertility complications, including post-UAE amenorrhea (affecting < 5% of patients), obstetric risks such as preterm delivery, cesarean section, and postpartum hemorrhage, and potential endometrial alterations [11]. However, some European studies suggest that using carefully calibrated 500–700 µm particles with slow, controlled injection minimizes non-target embolization by preventing passage through utero-ovarian anastomoses, thereby preserving ovarian function [12–15].

Recent systematic reviews and meta-analyses, including two key 2024 studies, report that 40–69% of women desiring pregnancy achieve it post-UAE, with no significant adverse effects on ovarian reserve markers [16–19]. Notably, Peng et al. found no difference in live birth or miscarriage rates between UAE and myomectomy, while Tzanis et al. reported comparable pregnancy rates, providing level 1 evidence supporting UAE’s fertility outcomes [20, 21].

Despite these advances, UAE’s impact on fertility remains controversial, partly because women under 40 are rarely referred for UAE due to persistent misconceptions about its fertility implications, limiting its application in younger patients who could benefit most [22, 23]. This referral bias, favoring peri-menopausal women restricts the ability to assess UAE’s full potential in improving fertility outcomes for younger women with leiomyomas [24]. The lack of robust data on obstetric outcomes post-UAE further fuels hesitation among clinicians.

The aim of this study was to evaluate fertility outcomes after UAE for uterine leiomyomas in a tertiary care center.

## Materials and methods

### Study design and objectives

We conducted a single center retrospective study in an academic medical hospital assessing fertility after UAE in patients with symptomatic uterine leiomyoma. The study was approved by French ethics committee (SI number: 21.02572.000048; IDRCB: 2021-A01868-33) and has been registered in Clinicaltrials.gov (ID: NCT05271981). All participants were informed about the study and provided their oral consent to participate.

The primary objective of this study was to assess the rate of live births after UAE in patients with symptomatic uterine leiomyoma.

The secondary objective was to assess the rate of pregnancies and the rate of obstetric complications, including miscarriage rate, preterm delivery, mode of delivery, hemorrhage and other rare complications of pregnancy.

### Patient selection

**Fig. 1:**
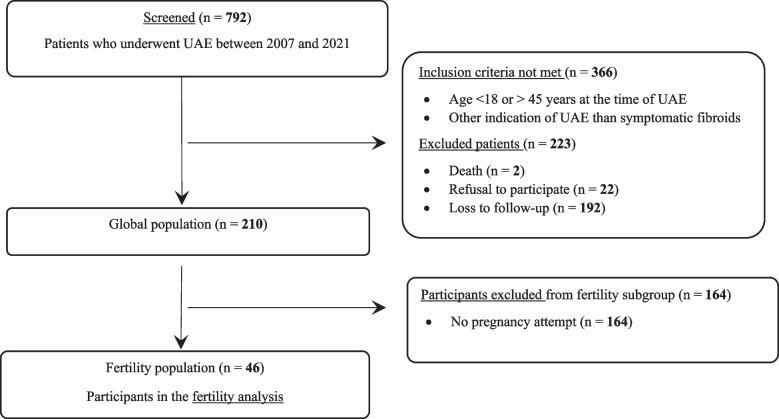
Flow chart

All consecutive patients who underwent UAE in our tertiary center between June 2007 and March 2021 were screened for inclusion. Inclusion criteria were age at UAE between 18 and 45 years included, symptomatic uterine leiomyoma as primary indication for UAE. Exclusion criteria were UAE for other primary condition and refusal to participate. Embolization was proposed preferentially to patients who had no desire for a subsequent pregnancy, guided by the surgical history and challenges. An information note and non-opposition statement were sent to all eligible patients. All patients willing to participate were included in the main analysis. The flow-chart is provided in Fig. 1.

### Embolization

**Fig. 2:**
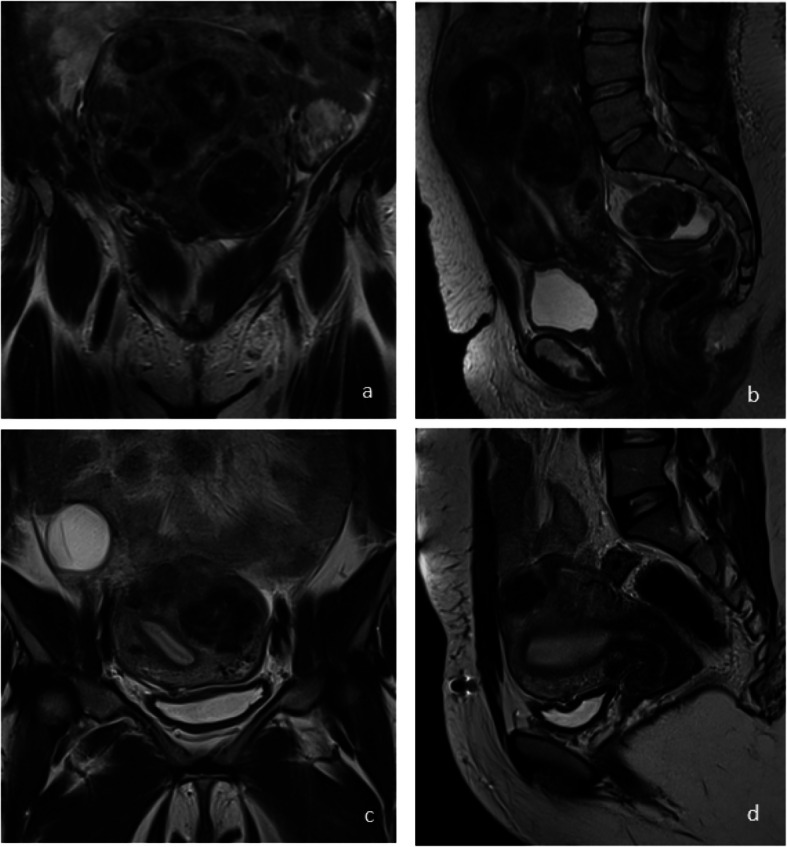
Pelvic MRI of an enlarged polymyomatous uterus ineligible for surgery. T2 weighted images, coronal (**a**, **c**) and sagittal (**b**, **d**) of pre-embolization (**a**, **b**) and four years post-embolization (**c**, **d**) MRI of woman embolized at the age of 42 for symptomatic fibroids. This patient had 3 pregnancies and 2 children with one cesarean and one voluntary termination of pregnancy, and a history of myomectomy before UAE. She did not have any pregnancy after UAE

Uterine artery embolization was performed following a consultation with the attending interventional radiologist in a dedicated department and after a multidisciplinary discussion including gynecologists. Every patient underwent a pelvic MRI to confirm the diagnosis, determine the number, localization, and size of uterine leiomyomas according to the FIGO classification [25] (Fig. 2). A consultation with the pain management team was conducted and analgesic and anti-inflammatory medications were prescribed before, during, and after UAE. Embolization was performed under local anesthesia usually through femoral artery puncture. All the embolized arteries were uterine. Patients were hospitalized at least one night after the intervention and were discharged with a prescription of analgesia.

### Follow-up

**Fig. 3:**
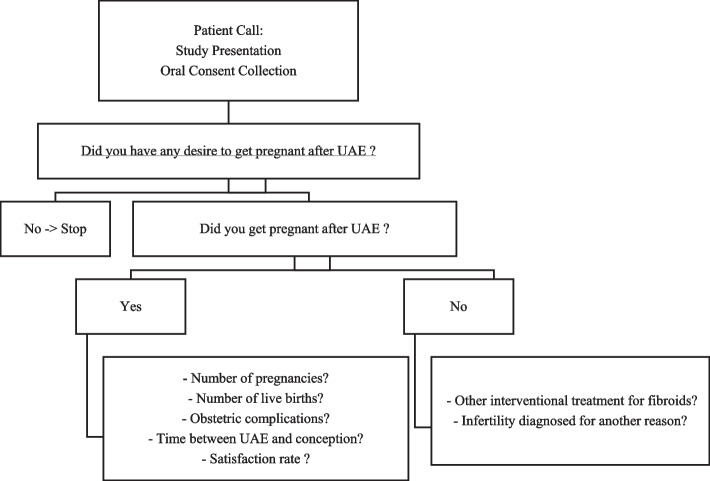
Telephone questionnaire on the desire to have children and achieving pregnancy

Patients included in the study received a subsequent phone call with one of the attending interventional radiologists or gynecologists to confirm their agreement and filled in a dedicated questionnaire on pregnancy wish and fertility outcome (Fig. 3). Pre-, intra-, and, when available, post-UAE data were collected from the patients' hospital records.

### Statistical analysis

The main analysis was based on the included population, i.e. all patients willing to participate. A subanalysis was conducted specifically for the fertility outcomes in patients who reported attempting pregnancy after UAE, defined as actively trying to conceive through unprotected intercourse or assisted reproductive technologies (ART), with the goal of achieving pregnancy. These patients were categorized as the “*fertility population.”*

All analysis were reported according to the STROBE statement [26]. Continuous variables were presented as means (± SD) for normally distributed data and otherwise as median and interquartile range (IQR).

Continuous variables were compared using two-sided t-tests. Categorical data are numbers and percentages. No replacement of missing data was performed. Analyses were done with SAS software, version 9.4 (SAS Institute). We deemed a *p* value less than 0.05 to be significant.

## Results

### Patient characteristics

A total of 792 consecutive women between June 2007 and March 2021 underwent UAE in our center and were screened for the present study. Two hundred and ten patients met the inclusion criteria and were included in the main analysis. Forty-six patients declared themselves to have attempted pregnancy after UAE and were included in the fertility subgroup analysis (*N* = 46/210, 22%). They were defined as the fertility population. Patient characteristics are provided in Table 1.

**Table 1:** Characteristics and fertility outcomes of study population

Baseline participant characteristics	Study population, *N* = 46
Age, years​ (mean ± SD)	40.39 ± 3.87​
History of pregnancy before UAE​ (N, %)	34/46 (74%)
Women with children before UAE​ (N, %)	25/46 (54%)
Uterus volume before UAE, cm^3^ (mean ± SD)	135.23 ± 33. 3
Dominant fibroid size before UAE, mm (mean ± SD)	66.3 ± 31.6​
BMI​ (mean ± SD)	​27,28 ± 5.64
Past gynecological history (N, %)​	
*Cesarean*	7/46 (15%)​
*Myomectomy*	20/46 (43%)​
*Miscarriage*	12/46 (26%)​
Other*	10/46 (22%)​
Symptoms before UAE (N, %):	
*Meno-metrorrhagia*	40/46 (87%)​
*Compressive symptoms*	11/46 (24%)​
*Pelvic pain*	27/46 (59%)​
*Anemia​*	15/46 (33%)​
**Fertility Outcomes**	**Study population, *N* = 46​**
Women with pregnancy beginning after UAE, N (%)	13/ 46 (28%)
*Number of pregnancy (mean number per woman)*	16 (1,2)
*Early miscarriage*	3/13 (23%)
*Late miscarriage*	0/13 (0%)
*Voluntary termination of pregnancy*	1/13 (8%)
Women with children born after UAE, N (%)	9/46 (20%)
*Number of live birth, N (mean number per woman)*	12 (1,3)
*Mean age at pregnancy, years (SD)*	37 ± 4.7
*Cesarean section*	9/9 (100%)
*Gestational hypertension*	1/9 (11%)
*Preeclampsia*	1/9 (11%)
*Gestational diabetes*	2/9 (22%)

^*^Including history of voluntary termination of pregnancy, tubal disease or plasty, ovarian cyst, ovarian failure, conization and preeclampsia

Mean age in the fertility population was 40 years (SD ± 3.9) at UAE and mean BMI was 27 (SD ± 5.6). All the women treated had several leiomyomas. UAE was performed using EmboSphere (Merit Medical), Embozen.

(Varian Medical) or Gelistaspon (Gelita) in respectively 42/46 (91%), 2/46 (4%) and 2/46 (4%) of the cases. Uterus’ mean size was 135 mm (SD ± 33) and the mean dominant uterine leiomyoma size was 66 mm (SD ± 32). The most common symptom before UAE was heavy menstrual bleeding (87%, *N* = 40/46). Other symptoms such as pain (*N* = 27/46, 59%), pelvic pressure (*N* = 11/46, 24%) and anemia (*N* = 15, 33%) were reported. Thirty-one patients (*N* = 31/46, 67%) tried oral hormonal therapy for leiomyomas before UAE. Past gynecological history included caesarian section (*N* = 7/46, 15%), myomectomy (*N* = 20/46, 43%), miscarriage (*N* = 19/46, 41.3%), and other (including history of Voluntary Termination of Pregnancy, tubal disease or plasty, ovarian cyst, ovarian failure, conization and pre-eclampsia, *N* = 10/46, 22%).

Regarding obstetrical history, 74% (*N* = 34/46) had at least one pregnancy prior to UAE with a total of 75 pregnancies and 54% (*N* = 25/46, 54%) had already at least 1 child. Nine patients (*N* = 9/46, 19.6%) had an ART history (1 intra-uterine insemination and 7 in vitro fertilization). Fourteen patients (*N* = 14/46, 38%) had an infertility diagnosed before embolization (1 ovarian failure, 5 pubertal infertilities, 3 male infertility). Mean follow-up after UAE was 9.2 years (SD ± 4.0 years).

### Fertility outcomes

Fertility outcomes in the whole population are presented Table 1. 28% (13/46) patients who attempted a pregnancy after UAE were pregnant at least once during follow-up. Age was independently associated with better fertility outcomes in univariate and multivariate analysis (*p* = 0.039).

The mean age at UAE in the successful live birth group was 37 years (SD ±—4.7) compared to 41 years (SD ± 3.3) in the live birth failure group (*p* = 0.039).

Out of the 13 pregnant women, 4 undergone assisted reproductive technology (ART). Nine women (69%) successfully gave birth, 3 (23%) experienced early miscarriages (with a miscarriage rate per pregnancy of 19%, *N* = 3/16), and 1 (8%) opted for voluntary termination of pregnancy. There was a total of 16 pregnancies in 9 women resulting in 12 live births: one patient had twins, and another patient had 3 children after UAE (in 3 different deliveries). Among the successful live birth group after UAE, all 9 patients underwent cesarean section (*N* = 9/9, 100%). Six of these patients had a scarred uterus, two patients due to a previous cesarean section before embolization, and four patients due to a history of myomectomy. Non-fatal obstetrical complications occurred in 6 patients (*N* = 6/9, 67%): gestational hypertension (*N* = 1/9, 11%), preeclampsia (*N* = 1/9, 11%), postpartum hemorrhage (*N* = 2/9, 22%) and gestational diabetes (*N* = 2/9, 22%). There were no cases of fetal death in utero or placenta previa. Among the 10 infertile patients in the population, the diagnosis was done before UAE in half. Detailed description of patients included in fertility analysis is presented Table 2.

**Table 2:** Detailed description of patient included in fertility analysis (patient underlined in grey successfully achieved a pregnancy after UAE)

At UAE time							After UAE			
Patient ID	Year of UAE	Age (years)	History of myomectomy by laparotomy	Gravidity (G) and parity (P)	Assisted reproductive technology before UAE (yes/no)	Uterine volume (cm3)	Pregnancy (yes/no, N)	Time from UAE to first pregnancy (months)	Live-born child (N)	UAE Satisfaction score (/100)
1	2020	34	*yes*	G1 P1	*no*	806	*no*			100
2	2020	38	*yes*	G1 P1	*yes*	Missing data	*no*			20
3	2020	41	*no*	G2 P2	*no*	241	*yes*	12	0	100
4	2020	45	*no*	G7 P4	*no*	Missing data	*no*			80
5	2019	35	*no*	G3 P1	*no*	288	*yes*	24	1	50
6	2019	38	*yes*	G2 P0	*no*	Missing data	*no*			50
7	2019	39	*no*	G4 P4	*no*	522	*no*			50
8	2019	42	*yes*	G3 P2	*no*	1602	*no*			DM
9	2019	43	*no*	G1 P0	*no*	1797	*no*			100
10	2018	41	*yes*	G0 P0	*no*	284	*no*			100
11	2018	42	*yes*	G1 P1	*yes*	Missing data	*yes*	Missing data	1	Missing data
12	2017	42	*yes*	G0 P0	*yes*	Missing data	*no*			100
13	2017	45	*yes*	G0 P0	*no*	806	*no*			100
14	2016	41	*no*	G4 P1	*yes*	473	*no*			100
15	2016	45	*yes*	G1 P1	*no*	892	*no*			70
16	2015	39	*no*	G2 P0	*no*	315	*no*			100
17	2015	39	*yes*	G3 P0	*no*	796	*yes*	24	1	Missing data
18	2015	42	*no*	G1 P1	*no*	595	*yes*	24	0	10
19	2015	45	*no*	G1P1	*no*	Missing data	*yes*	1	0	100
20	2014	33	*yes*	G1 P1	*no*	372	*yes (2)*	40	1	80
21	2014	38	*no*	G4 P0	*no*	1103	*no*			100
22	2014	42	*yes*	G0 P0	*no*	1034	*no*			100
23	2013	36	*yes*	G2 P0	*yes*	1492	*no*			60
24	2013	37	*yes*	G2 P0	*no*	1379	*no*			Missing data
25	2013	37	*no*	G1 P1	*no*	126	*no*			50
26	2013	39	*no*	G2 P2	*no*	1103	*no*			75
27	2013	41	*yes*	G0 P0	*no*	1048	*yes*	24	2	100
28	2013	44	*no*	G1 P1	*no*	833	*no*			50
29	2012	42	*yes*	G2 P1	*no*	879	*no*			50
30	2012	45	*no*	G2 P2	*no*	247	*yes*	60	1	100
31	2012	45	*no*	G1 P1	*no*	795	*yes*	Missing data	0	100
32	2011	39	*no*	G2 P0	*no*	867	*no*			50
33	2011	43	*no*	G5 P3	*no*	532	*no*			Missing data
34	2011	45	*no*	G3 P2	*no*	938	*no*			75
35	2010	34	*no*	G0 P0	*no*	512	*yes*	1	1	100
36	2010	35	*no*	G3 P2	*yes*	63	*yes*	24	1	100
37	2010	41	*no*	G0 P0	*yes*	196	*no*			80
38	2010	45	*no*	G1 P1	*no*	648	*no*			100
39	2009	38	*yes*	G2 P0	*no*	368	*no*			Missing data
40	2009	44	*no*	G0 P0	*no*	728	*no*			Missing data
41	2008	34	*no*	G0 P0	*no*	1244	*no*			80
42	2008	41	*yes*	G0 P0	*yes*	924	*no*			50
43	2008	43	*no*	G3 P1	*no*	641	*no*			100
44	2008	45	*yes*	G0 P0	*yes*	885	*no*			100
45	2007	31	*no*	G1 P1	*no*	262	*yes (3)*	11	3	50
46	2007	45	*yes*	G0 P0	*no*	1061	*no*			100

### General outcomes and follow-up in the fertility population

After UAE, only one patient (*N* = 1/46, 2%) reported pelvic pain, and one patient (*N* = 1/46, 2%) reported heavy menstrual bleeding. The satisfaction rate regarding leiomyoma -related symptoms is on average 72%. Seventy percent of patients (*N* = 32/46) were fully treated after UAE and did not require any reintervention for leiomyoma -related symptoms. One patient (*N* = 1/46, 2%) had a second UAE, 3 patients (*N* = 3/46, 6%) a hysterectomy, and 5 patients (*N* = 5/46, 11%) a myomectomy. No major complications were reported after UAE.

## Discussion

The present study brings new data from a cohort of patients who attempted pregnancy after UAE. A third of women in our population were able to become pregnant after UAE, despite a mean age of 40 years. This result is encouraging compared to the general population where the chance of conceiving is around 40% at the age of 40 and even lower in patients with fibroids [3, 27]. This number is satisfactory given the history of patients with previous myomectomy, miscarriage and other morbidities. Also, previous obstetrical history, with most patients (50%) diagnosed with an infertility diagnosed before embolization.

Patients did not present with a higher rate of miscarriage (19%) or obstetrical complications when confronted to the general population where the miscarriage rate is about 27% at the age of 40 [27] and compared to published data [19, 28]. The high rate of cesarean could be independently discussed: studies show that women with large leiomyomas have a significantly increased cesarean section rate [29]. A recent meta-analysis found a cesarean delivery rate in the leiomyoma group of 60% compared with 39% for the no- leiomyoma group [30].

Several retrospective studies confirm that patients successfully conceive and deliver following embolization for postpartum hemorrhage [31–33]. However, multiple medical societies (CNGOF [34] in France, ACOG in USA [35], CIRSE [15]) still do not recommend embolization as a first-line treatment for patients wishing to preserve fertility when treating symptomatic uterine leiomyomas. For example, recent CNGOF menorrhagia guidelines suggest the following approach, that consists in “proposing, for women wishing to preserve their fertility, both uterine artery embolization or myomectomy and inform them about the uncertainties regarding future fertility and the higher risk of miscarriage after embolization” (*weak recommendation, low-quality evidence*) [36]. Although UAE is an effective and less invasive treatment for symptomatic leiomyomas, with level 1 evidence supporting comparable pregnancy outcomes to myomectomy, its adoption is hampered by referral biases favoring older women, outdated clinical guidelines, and difficulties in studying pregnancy due to multifactorial influences and lower baseline fertility in women with leiomyomas [19].

The potential impact of UAE on fertility relies on several hypothetical mechanisms, including the alteration of endometrial quality due to changes in uterine mucosal vascularization and the reduction of ovarian blood supply through anastomoses between uterine and ovarian arteries, leading to impaired follicular development and ovulation quality, and possibly premature ovarian failure [37]. In this study, utero-ovarian anastomoses were not systematically evaluated, but potential post-procedural menopause was monitored. Despite some missing data, no patients reported this condition. Including biological follow-up for the subgroup of patients desiring pregnancy could be valuable.

Approximately twenty studies evaluating fertility and obstetrical complications after uterine artery embolization have been published since 2000, primarily retrospective cohort studies. Only one randomized controlled trial, published in 2008 [38], compared embolization and myomectomy in 127 patients, finding a pregnancy rate of 50% in the embolization group and 78% in the myomectomy group (though not statistically significant). The miscarriage rate was 64% in the embolization group and 23% in the myomectomy group (*p* < 0.05). However, this trial has several biases, as its initial design was not intended for studying fertility, a short duration of the follow-up (mean of 24.9 months) and an unequal number of patients who tried to conceive in each group. The miscarriage rate found is the highest in literature, with studies including more patients reporting miscarriage rates between 13 and 56% [19, 39–41]. These rates need to be interpreted considering that the average age of patients achieving pregnancy in these studies was 37 years.

A literature review published in 2020 estimates a pregnancy rate of 38.3% after embolization [28]. Uterine artery embolization may also contribute to other obstetrical complications such as prematurity, postpartum hemorrhage, primarily due to uterine atony secondary to altered myometrial vascularization or malplacentation (accreta, previa), but this was not observed in our study [10, 42].

Several limitations within this study should be discussed. Firstly, this is a retrospective study. This leads to a certain number of people being lost to follow-up and a recall bias is possible when interviewing patients and some information remains missing. Secondly, ovarian function was not evaluated in our patients. However, a recent study comparing serum levels of reproductive hormones (FSH, LH, E2, AMH) and ovarian reserve indicators (antral follicle count and, ovarian volume) in 87 women undergoing UAE versus 87 myomectomies show no significant differences at any point up to 36 months post-treatment [43]. Similar results were found in several other studies, which show that there are also direct and independent impacts of leiomyomas on fertility, linked to the difficulty of implantation of the embryo [30]. Thirdly, at the time of inclusion, UAE was not recommended for patients desiring pregnancy. This created a selection bias toward this therapeutic modality, even though a small percentage of them later expressed a desire for pregnancy retrospectively. It should also be noted that in this context, the treated patients, despite their young age, were those for whom surgery was considered high-risk due to their medical history, particularly surgical history, or the distribution and number of leiomyomas. Developing stringent selection criteria would be crucial to identify the profiles most likely to benefit from UAE without compromising their reproductive goals. In this context, a multidisciplinary team management with gynecologists, radiologists, endocrinologists, and fertility specialists is essential to ensure holistic care. Comprehensive registries tracking of fertility outcomes and large scale randomized trials with standardized fertility assessment before and after treatment required to provide robust evidence.

## Conclusion

UAE may be a fertility-preserving option in women with symptomatic leiomyomas, offering a highly effective and less invasive alternative, especially for those with complex gynecological histories where surgery poses significant risks. While age and fibroids impact fertility, UAE’s proven success calls for updated clinical guidelines to reflect modern data, with further studies needed to reinforce these promising outcomes.

## Data Availability

The datasets used and/or analyzed during the current study are available from the corresponding author on reasonable request.

## Abbreviations

ART: Assisted Reproductive Technology
CIRSE: Cardiovascular and Interventional Radiological Society of Europe
CNGOF: National College of Gynecologists and Obstetricians
FIGO: International Federation of Gynecology and Obstetrics
HIFU: High-Intensity Focused Ultrasound
MRI: Magnetic Resonance Imaging
UAE: Uterine Artery Embolization
UK: United Kingdom
